# Surgical Suggestions

**Published:** 1915-09

**Authors:** 


### SURGICAL SUGGESTIONS.

In surgery, as in internal medicine, the thermometer is an indispensable instrument of diagnosis. The surgeon who, without examining his patient, attributes an unexpected post-operative rise of temperature to "the hot weather," "constipation" or "too much company" may find it difficult to explain later why he did not at once recognize the wound infection, pneumonia, cystitis or other complication that had developed.

---

There is a comparatively rare type of pelvic infection in women in which the pus points posteriorly through the sacro-sciatic foramen. Such a condition may be suspected if, after confinement, for example, an irregular fever is associated with pain in the lower lumbar and sacro-ilias regions, perhaps radiating into one or both thighs. Such an abscess may not be recognizable by vaginal or rectal palpation.—American Journal of Surgery.

